# Facility-level conditions leading to higher reach: a configurational analysis of national VA weight management programming

**DOI:** 10.1186/s12913-021-06774-w

**Published:** 2021-08-11

**Authors:** Edward J. Miech, Michelle B. Freitag, Richard R. Evans, Jennifer A. Burns, Wyndy L. Wiitala, Ann Annis, Susan D. Raffa, Stephanie A. Spohr, Laura J. Damschroder

**Affiliations:** 1 Veterans Affairs Center for Health Information & Communication, VA EXTEND QUERI, Roudebush VA Medical Center, Indianapolis, USA; 2 Veterans Affairs Center for Clinical Management Research, VA Ann Arbor Healthcare System, Michigan Ann Arbor, USA; 3 National Center for Health Promotion and Disease Prevention, Veterans Health Administration, Durham, North Carolina USA; 4 Department of Psychiatry & Behavioral Sciences, Duke University School of Medicine, Durham, North Carolina USA

**Keywords:** Obesity, Program evaluation, Implementation outcomes, Reach, Veterans, Local context, Coincidence analysis

## Abstract

**Background:**

While the Veterans Health Administration (VHA) MOVE! weight management program is effective in helping patients lose weight and is available at every VHA medical center across the United States, reaching patients to engage them in treatment remains a challenge. Facility-based MOVE! programs vary in structures, processes of programming, and levels of reach, with no single factor explaining variation in reach. Configurational analysis, based on Boolean algebra and set theory, represents a mathematical approach to data analysis well-suited for discerning how conditions interact and identifying multiple pathways leading to the same outcome. We applied configurational analysis to identify facility-level obesity treatment program arrangements that directly linked to higher reach.

**Methods:**

A national survey was fielded in March 2017 to elicit information about more than 75 different components of obesity treatment programming in all VHA medical centers. This survey data was linked to reach scores available through administrative data. Reach scores were calculated by dividing the total number of Veterans who are candidates for obesity treatment by the number of “new” MOVE! visits in 2017 for each program and then multiplied by 1000. Programs with the top 40 % highest reach scores (*n* = 51) were compared to those in the lowest 40 % (*n* = 51). Configurational analysis was applied to identify specific combinations of conditions linked to reach rates.

**Results:**

One hundred twenty-seven MOVE! program representatives responded to the survey and had complete reach data. The final solution consisted of 5 distinct pathways comprising combinations of program components related to pharmacotherapy, bariatric surgery, and comprehensive lifestyle intervention; 3 of the 5 pathways depended on the size/complexity of medical center. The 5 pathways explained 78 % (40/51) of the facilities in the higher-reach group with 85 % consistency (40/47).

**Conclusions:**

Specific combinations of facility-level conditions identified through configurational analysis uniquely distinguished facilities with higher reach from those with lower reach. Solutions demonstrated the importance of how local context plus specific program components linked together to account for a key implementation outcome. These findings will guide system recommendations about optimal program structures to maximize reach to patients who would benefit from obesity treatment such as the MOVE! program.

**Supplementary Information:**

The online version contains supplementary material available at 10.1186/s12913-021-06774-w.

## Background

Obesity poses a major challenge to healthcare systems, including the Veterans Health Administration (VHA) [1, 2]. Obesity is a well-established risk factor associated with increased morbidity in diabetes and hypertension in particular as well as increased mortality overall [3, 4]. Behavioral weight loss programs, pharmacotherapies, and bariatric surgery are effective treatments for obesity [5].

Recent estimates of the prevalence of obesity among Veterans receiving care in VHA are over 40 % [1]. Like many large health systems seeking to address the obesity epidemic, the Veterans Health Administration (VHA) healthcare system offers several evidence-based weight management options for patients, including a system-wide comprehensive lifestyle intervention (CLI) program, pharmacotherapies, and bariatric surgery [2]. Of these, the MOVE! Weight Management Program, the VHA CLI, is the most commonly available: on average, more than 100,000 Veterans participate annually in MOVE!, compared to about 2500 Veterans receiving weight loss medications and a few hundred Veterans who undergo bariatric surgery [2, 6]. For any treatment program to have meaningful population-level impact, programs must successfully reach patients to engage them in treatment [7]. Reach is an individual-level measure that refers to the percentage of eligible patients who receive a program [7]. Overall reach to patients who would benefit from obesity treatment in VHA remains suboptimal, as is the case with most health systems [8, 9].

Reach is a critical component of program evaluation. As Glasgow and colleagues emphasize, “understanding the degree to which a program reaches those in need is vital” [7]. However, achieving high reach in practice can be difficult, especially with comprehensive behavioral interventions. For example, a 2017 study on the implementation of the Diabetes Prevention Program in VHA – a complex lifestyle intervention – found that the biggest challenges to implementation were barriers related to reach [8]. The aim of this study was to identify characteristics of medical center-based obesity treatment program arrangements that lead to higher reach to patients who would benefit from obesity treatment. This program evaluation qualified as non-research quality improvement activity conducted under the authority of VHA operations.

## Methods

The National Center for Health Promotion and Disease Prevention (NCP) leads weight management policy and provides support to MOVE! weight management coordinators in VHA. In 2017, NCP collaborated with VHA’s Healthcare Analysis and Information Group (HAIG) to field a comprehensive survey of weight management programming across VHA. The operational survey was administered to 140 medical centers and achieved a 100 % response rate. It provides standardized and detailed information about local obesity treatment programming at each of the 140 facilities. Applying configurational analysis methods offers a unique opportunity to explore the link between reach and these facility-level treatment arrangements, including bariatric surgery, pharmacotherapy, and a wide range of treatment options within the MOVE! CLI program.

### Outcome

Our primary outcome was reach, which broadly defined is the extent to which a treatment reaches the population of interest [10]. In this analysis, reach scores were calculated using treatment data from the VHA Corporate Data Warehouse. For each facility, the score represented the total number of patients with at least one MOVE! visit in 2017 divided by the total number of Veterans at that facility who would benefit from MOVE! in 2017, then multiplying that number by 1000. Of the 140 facilities that responded to the HAIG survey, 127 (91 %) had sufficiently complete reach data necessary to calculate the reach outcome. We next calculated quintiles of reach. Facilities with reach scores within the upper two quintiles (i.e., the top 40 %, *n* = 51) and facilities within the bottom two quintiles (*n* = 51) were included in the analyses. Facilities with reach within the middle quintile (*n* = 25) were dropped from the analysis to build in a meaningful gap between the higher reach (i.e., upper 40 %) and lower reach (i.e., bottom 40 %) facilities; 102 facilities were in the analytic sample.

### Configurational analysis

Configurational analysis draws upon Boolean algebra and set theory to offer a formal, case-based and mathematical approach to cross-case analysis that identifies a “minimal theory,” i.e., a crucial set of difference-making combinations that uniquely distinguish one group of cases with an outcome of interest from another group without that same outcome [11]. The analytic objective of configurational analysis is to identify necessary and sufficient conditions for an outcome to occur, a fundamentally different search target than those used in correlation-based methods [11–13]. Configurational methods expressly allow for causal complexity (when several conditions must jointly appear for an outcome to occur) as well as equifinality (when multiple pathways lead to the same outcome), making it well-suited for discerning different solution pathways related to local context. Configurational analysis is one method within a broader family of configurational comparative methods, which include Qualitative Comparative Analysis and more recently Coincidence Analysis. These methods operate within a different paradigm than traditional methods like logistic regression or qualitative research, and their findings can complement those generated by other approaches [14].

Configurational methods have been applied in political science and sociology since the 1980s and are increasingly being used in health services research. A study by Kahwati et al., for example, used configurational methods to identify facility structure, policies, and processes related to the VA MOVE! program distinguishing 11 higher-performing facilities from 11 lower-performing facilities [15]. More recently, a 2019 Cochrane Review prominently used configurational comparative methods to identify conditions aligned with successful implementation of school-based interventions for asthma self-management [16]; a new comprehensive 2020 handbook on implementation science dedicated a complete chapter to configurational comparative methods [17]; and researchers have published numerous articles featuring configurational methods in prominent health services journals in 2020, including *BMC Health Services Research* [18–22].

### Creating the analytic dataset

To create the analytic dataset for the configurational analysis we merged two different datasets. The first was the 2017 HAIG survey of facility-level weight management programs, which had 78 items. The HAIG survey achieved a 100 % response rate, with all 140 medical centers responding. (For examples of items from the HAIG Survey, see Additional File 1).

The second dataset was FY17 facility-level weight management outcome data from the VHA Corporate Data Warehouse; 127 VA medical centers had sufficiently complete facility-level weight outcome data.

In the merged dataset of 127 facilities, we added “facility complexity level” as an additional potential explanatory factor. “Complexity level” is a preexisting operational designation within VHA where each facility is assigned one of five complexity levels based on patient characteristics, clinical services offered, educational and research missions, and administrative complexity [23, 24]. At the high end of the “complexity” continuum are urban medical centers with high overall patient volume, large numbers of high risk patients, complex clinical programs, and large research and teaching programs. At the low end are smaller facilities with low overall patient volume, smaller numbers of high risk patients, few (if any) complex clinical programs, and modest (if any) research and teaching programs [23, 24]. To reduce dimensionality, we further simplified the original five-level designation to three categories based on overall patient volume: high complexity level (VHA complexity level 1a, high patient volume); medium complexity level (VHA complexity levels 1b, 1c, and 2, medium and medium-high patient volumes); and low complexity level (VHA complexity level 3, low patient volume).

### Data reduction

The merged dataset comprised over 30 factors, many of which were multi-value, with multiple responses possible. For example, the survey item inquiring about the presence of a MOVE! Coordinator was scaled based on the level of time dedicated to the program, with 6 possible responses ranging from 0 to 100 %.

Our next step was to reduce the number of factors by creating three ordinal meta-factors that combined information from multiple individual factors from the original HAIG survey. For example, the role of MOVE! Coordinator is a key program leadership position. Three separate questions were asked related to the MOVE! Coordinator: the presence of a coordinator, the amount of time dedicated to MOVE!, and actual time spent on MOVE! each week. Table 1 lists these questions and the final integrated meta-factor used in the analysis. A similar process was used to create meta-factors indicating the degree of presence of a Physician Champion (another key role) and accessibility of weight loss medications. Two of the three meta-factors used 6-point ordinal scales (0 = None to 5 = Very high/highly active) to represent the “degree of presence” of a local MOVE! coordinator or a MOVE! Physician Champion. The third meta-factor consisted of a 4-point ordinal scale representing “accessibility” of weight-loss medications for MOVE! Participants (1 = medications not routinely considered to 4 = weight loss medications routinely considered with available prescriber); for the middle two values, our rationale for prioritizing routine consideration of weight loss medications over prescriber availability was that there are multiple options within the larger VHA system for getting access to medications if a local prescriber is not available.

**Table 1:** Meta-factors included in configurational analyses

Factor Name	Description
MOVE! Coordinator^a^	0 = None; 1 = “MINIMAL” (≤ 10 h/week); 2 = “MODERATE” (11–20 h/week); 3 = “HIGH” (21–30 h/week); 4 = “VERY HIGH_NOT FULLTIME” (< 100 % FTE; ≥31 h/week); 5 = “VERY HIGH_FULLTIME” (100 % FTE; ≥31 h/week)
Physician Champion^b^	0 = None; 1 = “NEAR ZERO” Not Funded_Minimal Activity (0 FTE; < 1 h/week); 2 = “FUNDED BUT NOT ACTIVE” Funded_Minimal Activity (10–25 % FTE; < 1 h/week); 3 = “UNFUNDED BUT ACTIVE” Not Funded_Moderate Activity (0 FTE; 1–5 h/week); 4 = “FUNDED AND ACTIVE” Funded_Moderate-High Activity (≥ 10 % FTE; 1–5 h/week); 5 = “HIGHLY ACTIVE” (≥ 6 h/week)
Access to Weight Loss Medications^c^	1 = Weight loss meds NOT routinely considered AND prescriber NOT available; 2 = Weight loss meds NOT routinely considered but prescriber is available; 3 = Weight loss meds routinely considered but prescriber NOT available; 4 = Weight loss meds routinely considered AND prescriber available

Legend

^a^Based on 3 survey questions: Q7. Does your facility currently have a permanent MOVE! Coordinator? (YES/NO); Q11. How much dedicated time is allotted for the MOVE! Coordinator role at your facility? (Select 1 of 6 listed options); and Q12. On average, how much time does the MOVE! Coordinator spend on the MOVE! Program each week? (Select 1 of 4 listed options)

^b^Based on 3 survey questions: Q13. Does your facility have a MOVE! Physician Champion? (YES/NO); Q16. How much dedicated time is allotted for the MOVE! Physician Champion role at your facility? (Select 1 of 6 listed options); and Q18. On average, how much time each week does the Physician Champion devote to the activities listed in the previous question? (Select 1 of 6 listed options)

^c^Based on 2 survey questions: Q23. Are weight loss medications routinely considered during treatment planning for MOVE! participants at your facility? (YES/NO) and Q24. Is a prescriber available to facilitate access to weight loss medications for patients participating in MOVE! (For example, is there a prescriber who works closely with the MOVE! program)? (YES/NO)

Next, we proceeded with data reduction by adopting a configurational approach to factor selection that has been described previously [18, 20, 21]. Briefly, we began by using the “minimally sufficient conditions” (i.e., msc) function within the R package “cna” [25] to look across all candidate factors for all 102 facilities to identify configurations of conditions with strong connections to the outcome condition (i.e., high reach). We considered all one-, two-, and three-condition configurations of conditions instantiated within our dataset that met the designated thresholds for consistency and coverage. Consistency is a metric that indicates how reliably a solution yields an outcome and is calculated as the number of cases with the solution configuration and the targeted outcome divided by the total number of cases with the solution configuration. Coverage is a metric that indicates how broadly a solution accounts for an outcome and is calculated as the number of cases with the configuration and the outcome present divided by the total number of cases with the outcome.

We then generated a “condition table” to list and organize the Boolean output. In a condition table, rows contain all configurations of conditions that meet a specified consistency level while column variables include outcome, conditions, consistency and coverage. We generated condition tables by specifying a consistency threshold of 100 % and a coverage score of at least 15 % to avoid overfitting. If no configurations met this dual threshold, we iteratively lowered the specified consistency level by 5 points (e.g., from 100 to 95 %, etc.) and repeated the process to generate a new condition table. We continued relaxing the consistency threshold until there were at least two potential configurations of facility-level conditions that met the specified consistency level and the ≥ 15 % coverage threshold. We then assessed all configurations that satisfied those thresholds.

Using this approach, we inductively analyzed the entire dataset and reduced the number of factors to those most likely to inform model development in the next steps of configurational analyses. We developed models by iteratively using model-building functions within the Coincidence Analysis (i.e., “cna”) software package in R [25]. We assessed models based on their overall consistency and coverage, as well as potential model ambiguity (when competing models explain the outcome equally well as reflected by similar consistency and coverage scores) [26]. After a preliminary model was identified, we optimized coverage by returning to the condition table and considered any additional configurations that met the consistency threshold and also covered ≥ 15 % of the cases where the outcome was present but not yet explained. We selected a final model based on the same criteria of overall consistency and coverage, with no model ambiguity. The Coincidence Analysis package (“cna”) in R, R (version 3.5.0), R Studio (version 1.1.383) and Microsoft Excel were used to support analyses.

## Results

Reach measures for the higher reach group ranged from 3.3 to 13.1 per 1000 Veterans; measures for the lower reach group ranged from 0 to 2.3 per 1000 Veterans.

During the data reduction process, four different categories of factors and ten different conditions were represented by the range of configurations that met our dual threshold criteria (see Table 2). The final solution model comprised conditions that explained 78 % (40/51) of the higher-reach facilities with 85% consistency (40/47) and consisted of five distinct pathways. Three of these five pathways were context-dependent: distinct pathways leading to higher reach, depended on whether the facility was high-, medium- or low-complexity. Multiple pathways included conditions that spanned across the MOVE! program, pharmacotherapies, and/or bariatric surgery; specific components within these treatment domains had to be jointly present to achieve higher reach to patients.

**Table 2:** List of factors included in configurational solution

Factor Name (Question number in HAIG survey)	Description
MOVE! Program Factors	
MOVE! Coordinator (See Table 1)	High degree of presence (more than 20 h per week is spent with the MOVE! program)
Be Active and MOVE!^a^ (Q21)	Be Active and MOVE! Programming is offered: 0 = No; 1 = Yes
Maintenance Program (Q21)	Maintenance programming is offered: 0 = No; 1 = Yes
Pharmacotherapy Factors	
Pharmacist Expertise (Q30)	Facility has a pharmacist(s) with expertise in weight management medications: 1 = Yes; 2 = No; 3 = Don’t Know
Pharmacotherapy offered in MOVE! (Q25)	Pharmacotherapy is considered during treatment planning for MOVE! 0 = No; 1 = Yes
Prescription Refill Restrictions (Q28)	Besides criteria for discontinuation of medication, are there are other restrictions or limits in place for refilling weight management medications: 1 = Yes; 2 = No; 3 = Don’t Know
Bariatric Surgery Factors	
Bariatric Surgery Option Offered (Q25)	Bariatric surgery is considered during treatment planning for MOVE! participants: 0 = No; 1 = Yes
Facility Context	
Facility Complexity Level^b^	Highest complexity (1a): Facilities with high volume, high risk patients, most complex clinical programs, and large research and teaching programs; High complexity (1b): Facilities with medium-high volume, high risk patients, many complex clinical programs, and medium-large research and teaching programs; Mid-High complexity (1c): Facilities with medium-high volume, medium risk patients, some complex clinical programs, and medium sized research and teaching programs; Medium complexity (2): Facilities with medium volume, low risk patients, few complex clinical programs, and small or no research and teaching programs; Low complexity (3): Facilities with low volume, low risk patients, few or no complex clinical programs, and small or no research and teaching programs

Legend

^a^Be Active and MOVE! is an ancillary physical activity module within the MOVE! Program

^b^Complexity score is based on size, academic affiliation, trauma center level, and service mix of the facility [24]

**Fig. 1:**
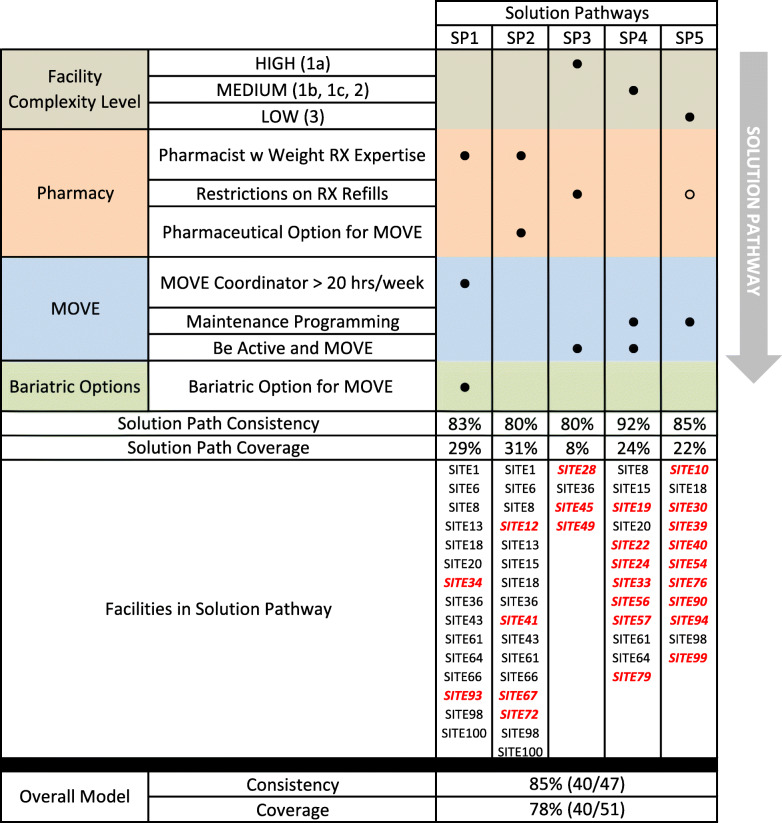
Summary of configurational findings for higher-reach VA facilities

Figure 1 provides a visual depiction of configurational results. First, in the top section of the figure, conditions (i.e., when factors take on specific values) are listed in rows. Three conditions relate to context of the medical center (high, medium, or low complexity). Next, three conditions relate to the MOVE! comprehensive lifestyle intervention, three conditions relate to pharmacotherapy, and one condition relates to bariatric surgery. Each of the sections are color-coded. Columns on the right indicate which conditions are included in each of the five pathways leading to higher reach scores. Black circles indicate the *presence* of the respective condition, an open circle indicates the *absence* of the condition, and blank space indicates that the condition is not part of that pathway.

The five pathways listed in Fig. 1 are indicated by column. All conditions with circles within that column are part of the pathway and jointly lead to higher reach. Filled circles indicate the presence of a condition, whereas empty circles indicate absence. For example, Solution Pathway 1 (SP1) indicates that the joint presence of a pharmacist with expertise in weight loss medications AND the presence of a local MOVE! coordinator who works with MOVE! more than 20 h per week AND programs where bariatric surgery is considered as part of treatment planning for MOVE! patients consistently lead to higher reach. SP3-SP5 each have a condition related to facility complexity. While the *absence* of refill restrictions is part of the solution for low complexity facilities (SP5), the *presence* of refill restrictions is on the solution pathway for high complexity facilities (SP3).

Figure 1 lists consistency for each of the pathways and ranges from 80 % (SP2-SP3) to 92 % (SP4), indicating high reliability for all pathways. Raw coverage indicates the percentage of high reach sites that are included in each solution pathway; raw coverage ranged from 8 % (SP3) to 31 % (SP2). At the bottom of Fig. 1, individual sites are listed by study identification number. Facilities listed in red italics represent cases that are uniquely covered by that solution pathway. For example, two of the 15 sites covered by SP1 are listed in red italics, indicating that those cases were explained only by that solution pathway and not by any of the other four pathways. A total of 18 sites have the combination of conditions for SP1; 15 sites are higher reach and three sites (not listed in figure) are lower reach. Thus, consistency is 83 % of all cases with the solution path (15/18) and raw coverage is 29 % of all cases with higher reach (15/51). Figure 1 also lists overall model consistency and coverage scores that is an aggregated score across all five solution paths. The overall model had a consistency score of 85 % (40/47) and a coverage score of 78 % (40/51).

## Discussion

No single condition explained the 51 facilities with the higher-reach outcome; rather, *combinations* of specific conditions consistently and uniquely distinguished higher reach facilities from lower reach facilities. The final model comprised five solution pathways that each led to higher reach, indicating that multiple combinations can lead to the same outcome; this demonstrates equifinality in solutions. The final model included 78 % of the higher reach sites with 85 % consistency indicating a meaningful and reliable solution.

VHA convened a State-of-the-Art (SOTA) conference in 2016 to address the epidemic of obesity in the U.S., including among Veterans. Recommendations included the need for integrated multi-component treatment [2], requiring coordination across domains of treatment including bariatric surgery, pharmacotherapy, and lifestyle intervention [27] to engage patients in treatments tailored to meet their needs. Our results add credence to this recommendation: all but one solution pathway involved at least two of three domains of care and one pathway included conditions across all three. Thus, it is insufficient to offer only lifestyle intervention without the availability of pharmacotherapies, or pharmacotherapies without bariatric surgery options. Clinical practice guidelines for obesity treatment call for consideration of pharmacotherapy treatment and bariatric surgery concurrent to lifestyle intervention [28]. Within some VHA medical centers, patients participate in the MOVE! weight management program prior to bariatric surgery and may participate in maintenance programming post-surgery.

It is important to note that three of the solution pathways are context-based; the solution pathway depends on the complexity of the medical center. A key tenet within implementation science is that programs must be adapted to local conditions to optimize and sustain outcomes [29–31]. Within low complexity medical centers (smaller, often rural centers), having maintenance programming with no restrictions on weight loss medication refills led to higher reach; this pathway explained all 11 low complexity sites with higher reach in our dataset. For medium complexity centers, the “Be Active and MOVE!” (BAM) physical activity adjunct program combined with maintenance programming led to higher reach. The addition of BAM indicates an exceptionally expansive MOVE! program; only 23 % of all facilities reported offering BAM. For high complexity centers, the combination of BAM with restrictions on weight loss medication refills led to higher reach. The inclusion of the latter condition is a bit harder to understand; the presence of restrictions in a high complexity site may indicate active involvement of pharmacists in treatment or may be a proxy for another unmeasured factor.

### Limitations

This configurational analysis was conducted using a dataset comprised of program survey responses from all VHA medical centers, combined with administration data to compute reach scores. Though the dataset is large (*n* = 102 medical centers in the final dataset), our findings may not apply to settings outside the VHA. Information about weight management programming at each facility relied on self-reporting by VHA staff who may or may not have had close involvement with MOVE!, pharmacotherapy treatments, or bariatric surgery. Though coverage was high (78 %), 11 of the 51 higher reach facilities were not included in any of the five solution pathways, indicating a possible role for additional factors beyond those in the model.

## Conclusions

In this evaluation, configurational analyses revealed specific combinations of facility-level obesity treatment programming that consistently and uniquely distinguished higher-reach from lower-reach facilities. These findings represent Boolean conjunctions, where multiple conditions together yield an outcome. In health services research, configurational analysis appears to be well-suited to identify how the interplay of specific conditions leads to a complex phenomenon like higher reach within a large system of care.

These results also illustrate equifinality: there is more than one way to achieve higher reach to patients who may benefit from obesity treatment. In this respect, configurational analysis can help address a long-standing challenge in health services research: how to account for local context in cross-case analysis. Our solution for higher reach included three context-dependent pathways, which we could identify because configurational analysis has the capacity to model Boolean disjunctions, where different paths lead to the same outcome. As organizational context can play as large a role in determining outcomes as program components themselves [32, 33], the ability of our configurational analysis to detect one pathway for large urban medical centers and another pathway for smaller rural facilities underscores the potential contribution of this approach to the larger repertoire of evaluation strategies within health services research. Our findings will inform recommendations about optimal program structures for obesity treatment within VHA.

## Supplementary Information

**Additional file 1.** Examples of items from 2017 HAIG Survey.

## Data Availability

A de-identified version of the configurational data used in the analysis is available from the corresponding author on reasonable request.

## Abbreviations

BAM: Be Active and MOVE!
CLI: Comprehensive Lifestyle Intervention
HAIG: VHA Healthcare Analysis and Information Group
NCP: VHA National Center for Health Promotion and Disease Prevention
SOTA: State-of-the-Art
SP: Solution Pathway
VHA: Veterans Health Administration
