# Supplementary material for: Facility-level conditions leading to higher reach: a configurational analysis of national VA weight management programming
Source: BMC Health Serv Res. 2021 Aug 11;21:797. doi: 10.1186/s12913-021-06774-w (PMC8359110; doi:10.1186/s12913-021-06774-w)
Supplement: Supplementary file 1 — Additional file 1. Examples of items from 2017 HAIG Survey. [file 12913_2021_6774_MOESM1_ESM.pdf]

2017 Weight Management Care In VHA Survey

WHY SURVEY?

Forty two percent of Veterans treated in the Veterans Health Administration (VHA) are obese as defined by a body mass index (BMI) $\geq$30 and another 37% are overweight (25 $\leq$ BMI <30). Overall, 3.9 million Veterans treated in VHA are overweight or obese. Overweight and obesity are linked with increased prevalence of chronic health conditions, including type 2 diabetes, hypertension, dyslipidemia, osteoarthritis, and obstructive sleep apnea, as well as reduced quality of life and premature mortality. VHA provides evidence-based care for overweight and obesity in the form of the behavioral MOVE! Weight Management Program, FDA-approved weight loss medications, and surgical interventions. In order for Veterans to maximally benefit from these interventions, they must be integrated and coordinated at various levels of the organization. This survey is the first step to establishing an integrated, coordinated approach to weight management and obesity care in VHA.

SURVEY OBJECTIVE

- To collect baseline information about existing weight management services for Veterans receiving care at VHA facilities and characterize variability across VHA. This will inform efforts to more consistently align VA weight management services with the VA/DoD Clinical Practice Guideline for Screening and Management of Overweight and Obesity and generally accepted weight management standards of care.
    - Baseline information includes the following topics:
        - Weight Management Clinical Care Characteristics (range of weight management services, frequency of delivery, etc.)
        - Weight Management Committees
        - Weight Management Programming (structure, processes, staffing)
        - Processes for of Weight Management Care Options
        - Veteran Engagement in Weight Management Program Planning and Evaluation

Following the survey, specific goals for dissemination are as follows:

- To make that information <u>accessible to stakeholders</u> (including field-based program personnel as well as facility and Network leadership) for program evaluation, improvement, and sharing of strong practices;
- To make that information <u>accessible for national programming staff</u> to facilitate and optimize accurate reporting, planning, improvement, and oversight;
- <u>To foster networking</u> among program personnel and integration of treatment options across service lines by promoting programmatic excellence, programmatic consistency, and inter-professional collegiality.

Example Survey Items

Does your facility currently have a permanent MOVE! Coordinator?

( ) Yes
( ) No

If no,

a. Does your facility have an acting MOVE! Coordinator?
( ) Yes
( ) No

b. Is your facility actively recruiting for a MOVE! Coordinator?
( ) Yes
( ) No

On average, how much time does the MOVE! Coordinator spend on the MOVE! Program each week?

(Choose one)

( ) 0 to 10 hours
( ) 11 to 20 hours
( ) 21 to 30 hours
( ) 31 or > hours

How much dedicated time is allotted for the MOVE! Coordinator role at your facility?

( ) 0 FTEE
( ) 0.1 to 0.25 FTEE
( ) 0.26 to 0.50 FTEE
( ) 0.51 to 0.75 FTEE
( ) 0.76 to 0.99 FTEE
( ) 1.0 FTEE

FTEE	week
1.0	40 or more
.875	35
.750	30
.625	25
.500	20
.375	15
.250	10
.200	8
.175	7
.150	6
.125	5
.100	4
.075	3
.050	2
.025	1

Does your facility have a MOVE! Physician Champion?
( ) Yes
( ) No

On average, how much time each week does the Physician Champion devote to the activities listed in the previous question?
(Choose one)
( ) Less than 1 hour per week
( ) 1 to 5 hours per week
( ) 6 to 10 hours per week
( ) 11 to 15 hours per week
( ) 16 to 20 hours per week
( ) More than 20 hours per week

How much dedicated time is allotted for the MOVE! Physician Champion role at your facility?
(Choose one)
( ) 0 FTEE
( ) 0.1 to 0.25 FTEE
( ) 0.26 to 0.50 FTEE
( ) 0.51 to 0.75 FTEE
( ) 0.76 to 0.99 FTEE
( ) 1.0 FTEE

Which of the following types of MOVE! programming are offered at your facility?
(Check all that apply)
[ ] a. Orientation to MOVE! and other weight management services
[ ] b. MOVE! Group Sessions (series)
[ ] c. MOVE! Individual Sessions (series)
[ ] d. Individual consultation
[ ] e. Maintenance programming
[ ] f. TeleMOVE! (Home Telehealth)
[ ] g. MOVE! Telephone Lifestyle Coaching
[ ] h. Be Active and MOVE!
[ ] i. MOVE! Coach with Care
[ ] j. MOVE! Employee
[ ] k. MOVE! for Special Populations
[ ] l. None

Which of the following interventions are considered during treatment planning for MOVE! participants? ***(Check all that apply)***
[ ] Bariatric surgery
[ ] Intragastric balloon
[ ] Pharmacotherapy
[ ] Very low-calorie diet
[ ] Meal replacement interventions
[ ] Inpatient or residential treatment
[ ] Other

Besides criteria for discontinuation of medication, are there any other restrictions or limits in place for refilling weight management medications at your facility/HCS?
( ) Yes
( ) No
( ) Don't know

Does your facility/HCS have a pharmacist(s) with expertise in weight management medications?
( ) Yes
( ) No
( ) Don't know
